# Comparative Analysis of AI and Ophthalmologist Grading in Diabetic Retinopathy Detection

**DOI:** 10.3390/biomedicines14020290

**Published:** 2026-01-28

**Authors:** Patricio M. Aduriz-Lorenzo, Jyothsna Rajagopal, Pradeep Walia, Gh Mustuffa Khan, Harini Indusekar

**Affiliations:** 1 Quironsauld Hospital Costa Adeje, 38660 Adeje, Spain; 2 Artelus System Private Limited, Bengaluru 560102, India; j.rajagopal@artelus.ai (J.R.);

**Keywords:** diabetic retinopathy, diabetic macular edema, artificial intelligence, screening, DRISTi

## Abstract

**Background:** Diabetic retinopathy (DR) poses a significant global health challenge that needs scalable and efficient screening pathways beyond the current limitations of teleophthalmology. This study retrospectively evaluated the diagnostic performance of an artificial intelligence (AI) DRISTi system (Version 2.1) against ophthalmologist grading for more-than-mild diabetic retinopathy (mtmDR), vision-threatening diabetic retinopathy (vtDR), and diabetic macular edema (DME). **Methods:** The methods involved a retrospective, observational, non-interventional validation comparing the AI DRISTi system’s output to ophthalmologist grading on 739 colour fundus images acquired using Topcon NWC 400, CrystalVue NFC 600/700, Canon CR2/CR2 AF, and Zeiss VISUCAM 500 cameras. **Results:** Primary outcomes included sensitivity and specificity, with statistical analyses utilizing 2 × 2 contingency tables and 95% confidence intervals. The AI system achieved an accuracy of 93.36% (sensitivity 95.03%; specificity 92.90%) for mtmDR, 98.64% (sensitivity 96.92%; specificity 99.01%) for vtDR, and 97.97% (sensitivity 92.85%; specificity 98.88%) for DME. Performance was robust and consistent across all evaluated camera types. **Conclusions:** In conclusion, the AI DRISTi system (Version 2.1) demonstrates strong diagnostic performance for mtmDR, vtDR, and DME, comparable to leading commercial AI systems, from fundus photographs acquired across multiple camera platforms. This system holds significant promise as an adjunctive screening tool for large-scale DR screening programs, contributing to early detection, appropriate triage, and the prevention of vision loss in at-risk populations.

## 1. Introduction

Diabetes Mellitus is a significant public health challenge that currently affects an estimated 589 million adults, projected to increase to 853 million adults by 2050 [1]. Diabetic retinopathy (DR) is an end-organ sequel of diabetes that affects the blood vessels of the retina. Individuals with poor glycemic control, hypertension, hyperlipidemia, and long-standing diabetes are at increased risk of developing DR. The global prevalence of DR among people with diabetes is 22%, and it is the leading cause of preventable blindness in those 25 to 74 years of age [2]. DR typically progresses from non-proliferative diabetic retinopathy (NPDR), the early stage characterized by microaneurysms and hemorrhages, to proliferative diabetic retinopathy (PDR), the advanced stage marked by the formation of abnormal new vessels. PDR carries a high risk of vision-threatening complications, such as vitreous hemorrhage and tractional retinal detachment. As the disease progresses, associated diabetic macular edema (DME) may also develop and is characterized by fluid and hard exudates at the macula. The ETDRS severity scale, based on detailed colour fundus photography, is considered the gold standard for staging DR. The International Clinical Diabetic Retinopathy (ICDR) classification is widely adopted in clinical practice and screening programs [3]. It categorizes DR into stages of increasing severity: mild, moderate, and severe NPDR and PDR, making it more practical to identify patients requiring serial monitoring versus those requiring referral and treatment.

The early stages of DR are often asymptomatic, and patients may not experience vision loss until advanced disease has already developed. Routine screening is essential for early detection and treatment to prevent progression to irreversible blindness. Effective screening differentiates patients who require urgent ophthalmic referral from those who can be safely monitored. Patients with no or mild NPDR without DME have a very low risk of progression to vision-threatening disease, are termed non-referable DR, and can be re-screened after one to two years. Patients with moderate and severe NPDR, PDR, and DME are classified as referable DR due to a higher risk of vision loss [4]. Screening tools must demonstrate high sensitivity to detect referable DR, particularly for vision-threatening stages, while also being cost-effective, easy to administer, and widely scalable. In regions with few ophthalmologists, large-scale screening for DR is a challenge. Teleophthalmology involves trained technicians capturing fundus images in remote areas, which are then transmitted to ophthalmologists elsewhere for grading. Although this screening tool reduces travel costs, addresses specialist availability, and improves access, it still requires a human grader for each image, and the bottleneck of a limited number of ophthalmologists remains. Moreover, teleophthalmology workflows are often asynchronous, leading to delays between image acquisition, grading, and patient notification. As a result, many patients with referable DR are lost to follow-up, particularly in rural settings without permanent infrastructure. Thus, while telemedicine improves geographic access, it remains constrained by human resource limitations, time delays, attrition, and scalability issues [5,6].

The promising potential of AI-based screening tools can overcome these limitations. These tools offer instant point-of-care diagnosis and can rapidly screen large volumes. Trained on large fundus image datasets, these AI tools use CNN algorithms to identify signs of referable DR. Several such tools for DR screening are now commercially available after regulatory approval, including IDx-DR, EyeArt, SELENA, and Google’s ARDA system. These tools have already been deployed in primary care and DR screening programs in the United States, the UK, and Singapore, marking a significant step forward in DR screening. This study evaluates a similar AI system. In a previous study using Version 1 of this DRISTi AI system on 753 gradable colour fundus images, a sensitivity of 90%, a specificity of 81%, and an overall accuracy of 90% was observed when detecting any DR. In particular, the sensitivity for referable DR was 96% [7]. This study further evaluates the diagnostic performance of DRISTi Version 2.1 compared to an ophthalmologist’s grading for the detection of referable DR on images acquired from multiple cameras.

## 2. Materials and Methods

### 2.1. Study Design and Timeline

This is a retrospective, observational, non-interventional validation study to assess the diagnostic performance of an AI system in detecting DR, using the ophthalmologist’s grading as the reference standard. 

### 2.2. AI System, Dataset, and Image Acquisition

Our algorithm utilizes a convolutional neural network (CNN), specifically a transfer-learning architecture based on EfficientNetV2S, pre-trained on ImageNet, and fine-tuned for vtDR, mtmDR, and Non-Referable classification. It begins with an input shape of (500, 500, 3), allowing compatibility with pre-trained ImageNet weights from the EfficientNetV2S model available via TensorFlow Hub. The training dataset consisted of 70,000 images, with 36,743 images collected from public datasets (EyePACS and MESSIDOR) and 33,257 images collected in India during community screening programs. A stratified random split was implemented to preserve the class distribution, resulting in an 80% train and 20% validation split. The study dataset consisted of colour fundus images of patients presenting for DR screening, collected from multiple secondary and tertiary eye care hospitals and community screening programs over two years. The images were captured in both mydriatic and non-mydriatic states using Topcon NWC 400 (Topcon Corporation, Tokyo, Japan), CrystalVue NFC 600/700 (Crystalvue Medical Corporation, Taoyuan City, Taiwan), Canon CR2/CR2 AF (Canon Inc., Kawasaki, Japan) and Zeiss VISUCAM 500 (Carl Zeiss Meditec AG, Jena, Germany) cameras. No demographic or clinical variables were available because the dataset was de-identified and anonymized. The dataset comprised single-field images (primary and nasal fields). Low-quality images that were blurred, dark, or incomplete were excluded, and all inputs were standardised in colour (RGB) and resized to 500 × 500 pixels for model compatibility.

### 2.3. Sample Size Estimation

To determine the sample size, we calculated the prevalence of DR to be 12. 5% for any DR, aiming for a sensitivity of 95% and a precision of 4.75% [8]. Furthermore, a buffer of 5% was included to cover non-gradable images, resulting in an estimated sample size of 713 images. A total of 760 images were selected, distributed as follows: 437 with no apparent DR, 179 with mild NPDR, 56 with moderate NPDR, 48 with severe NPDR, and 40 with PDR. These included 112 images with DME. A total of 60% of the photos had no DR features, and 5% of the images were of poor quality (e.g., insufficient focus, under/overexposure, inadequate field coverage, lens smudges, scratches, or other artifacts).

### 2.4. Reference Standard

The reference standard was established by an ophthalmologist with more than 20 years of clinical experience. The images were classified according to the International Clinical Diabetic Retinopathy Severity Scale (ICDR).

The DRISTi AI system classified each image into two categories:

- More-than-mild diabetic retinopathy (mtmDR), positive or negative;
- Vision-threatening diabetic retinopathy (vtDR), positive or negative.

### 2.5. Definitions

mtmDR positive: Moderate NPDR, severe NPDR, proliferative DR, and/or DME surrogate markers (hard exudates within one optic disc diameter of the macula);

mtmDR negative: Only mild NPDR or no DR, without surrogate markers of DME;

vtDR positive: Severe NPDR, proliferative DR, and/or DME surrogate markers;

vtDR negative: Moderate NPDR or less, without DME surrogate markers.

### 2.6. Outcome Measures

The primary outcome is the sensitivity and specificity of the AI system for detecting eyes with mtmDR or vtDR compared to the reference standard. The secondary outcomes include the AI system’s sensitivity and specificity for identifying eyes with DME relative to the reference standard, as well as performance across different cameras.

### 2.7. Statistical Analysis

Statistical analyses were performed using Python 3.11.9 with the NumPy 1.24.3 library. The sensitivity (actual positive rate) and specificity (actual negative rate) of the AI algorithm were calculated using 2 × 2 contingency tables. Additional performance measures included PPV, representing the probability of disease with a positive value, and NPV, representing the probability of no disease with a negative value. Ninety-five percent confidence intervals (95% CI) were computed for sensitivity, specificity, PPV, and NPV. A receiver operating characteristic (ROC) curve was derived using sensitivity against the false positive rate across varying thresholds, and the area under the curve (AUC) was calculated to assess the discriminative capacity of the AI system between DR and non-DR cases.

### 2.8. Ethical Considerations

An Independent Ethics Committee approved the study, and a waiver of informed consent was granted due to a retrospective study design and a de-identified dataset, in accordance with the ICMR guidelines (ART01). The study protocol was registered with the CTRI under the number CTRI/2025/10/095888. The study was conducted in accordance with the International Conference on Harmonisation Good Clinical Practice (ICH-GCP). All personal data were removed before analysis, and each patient was assigned a unique study identifier.

### 2.9. Data Management

The validation dataset was independent of the dataset used to train and develop the AI system. Data were stored in a secure electronic database, with access restricted to authorized study personnel. Data transfers were encrypted to preserve confidentiality. Image processing and inference were performed on secure servers.

## 3. Results

**Figure 1:**
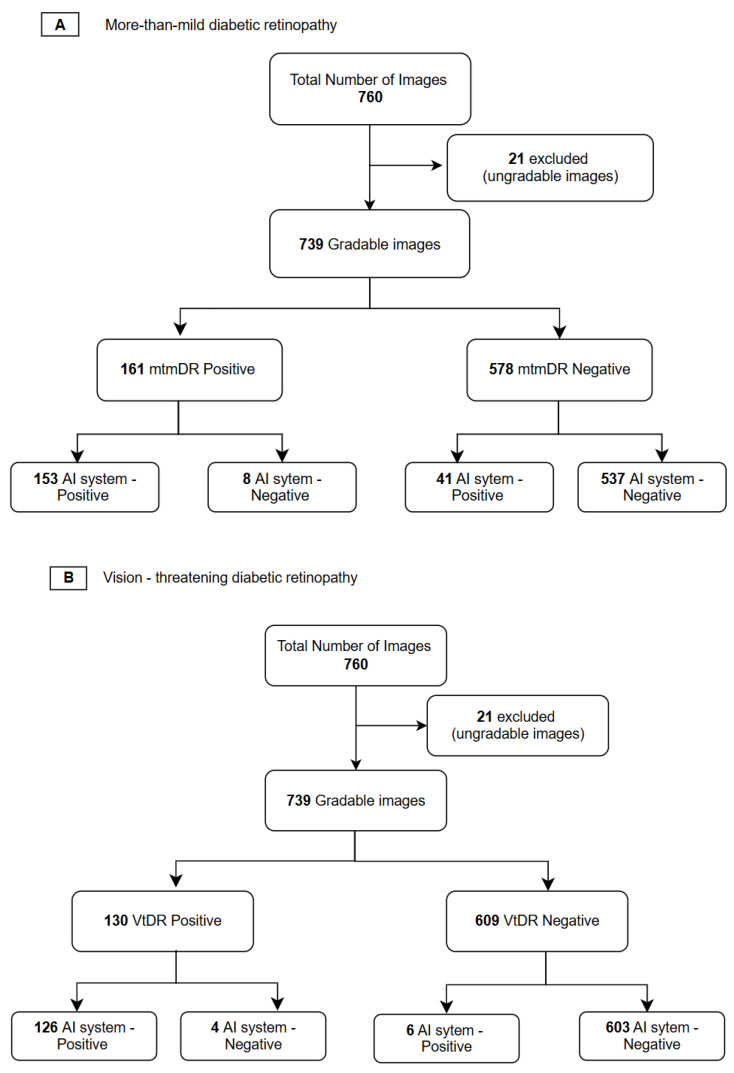
Representation of the STARD diagram of retinal image selection in the study.

**Figure 2:**
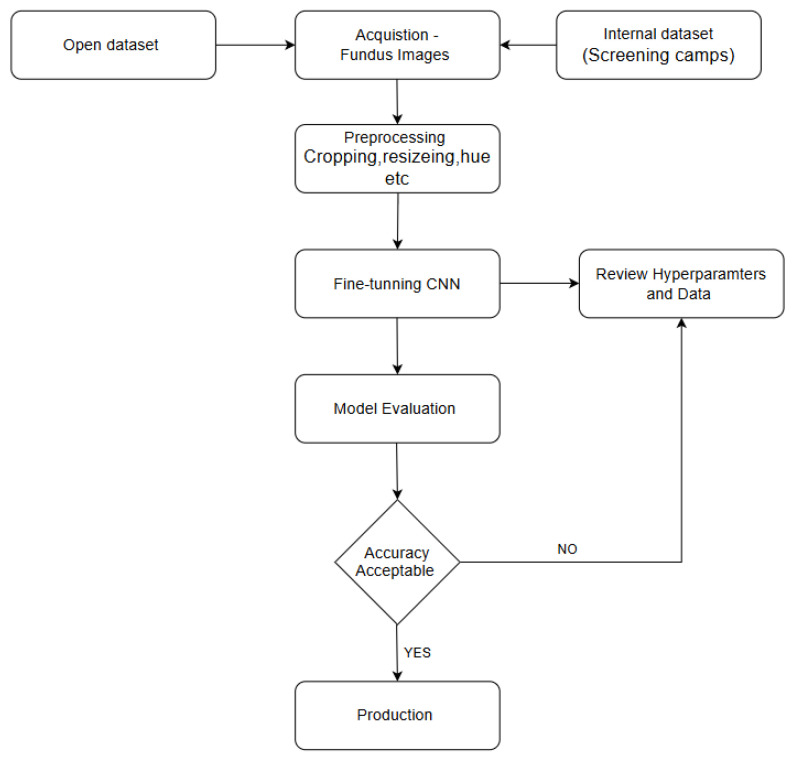
Flowchart of model development and evaluation pipeline.

Of the 760 images, 21 images were labelled as ungradable by the ophthalmologist and excluded from the analysis. The DRISTi AI system output for 739 images was compared with the ophthalmologist’s grading, as shown in Figure 1. The Figure 2 provides an overview of the model development and evaluation pipeline, illustrating the sequential steps involved in the workflow.

### 3.1. Performance of AI System on mtmDR Identification

For the subset of images with mtmDR, the AI system correctly identified 153 of the 161 images that were identified as mtmDR positive by the ophthalmologist, with a sensitivity of 95.03% (95% CI: 91.06–97.89%), and 537 of 578 images identified as mtmDR negative by the ophthalmologist, with a specificity of 92.91% (95% CI: 91.06–97.89%) Table 1.

**Figure 3:**
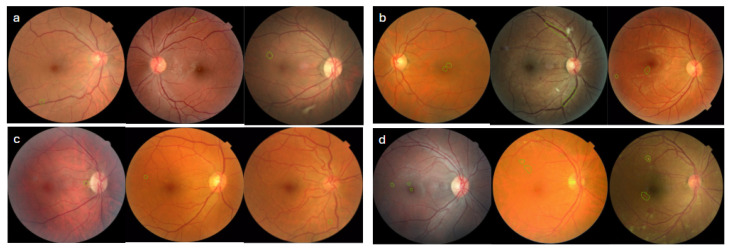
Panels (**a**–**d**) represent the following: (**a**) Images of false negative prediction from the mtmDR subset; (**b**) images of false positive prediction from the mtmDR subset; (**c**) images of false negative prediction from the vtDR subset; (**d**) images of false positive prediction from the vtDR subset.

In evaluating the eight images that resulted in false negative mtmDR classifications, four contained haemorrhages, that blended with the surrounding retinal background, reducing detectability. In three images, the model labelled the lesion as a microaneurysm, whereas the grader identified it as a hemorrhage (Figure 3a). The detailed PPV and NPV values are shown in Table 1.

Among the 41 images with false positive mtmDR results, the ophthalmologist graded 23 (56%) as mild NPDR. This disagreement largely stemmed from the difficulty in differentiating hemorrhages from microaneurysms and drusens from hard exudates. The remaining 18 images (44%) were graded as having no DR. Artefacts, pigment clumps, and densely tessellated retinal backgrounds likely contributed to the model’s misclassification of these images as hemorrhages (see representative examples in Figure 3b).

### 3.2. Performance of AI on vtDR

In evaluating the four images that resulted in false negative VTDR classifications. One image was graded by the clinician as showing venous beading. In the remaining three images, the model did not identify vtDR-associated pathological features (refer to Figure 3c).

For the subset of images with vtDR, the AI system correctly identified 126 of 130 images that were identified as vtDR positive by the ophthalmologist, with a sensitivity of 96.95 % (95% CI: 94.02–99.28%) and 603 of 609 images identified as negative for VtDR by the ophthalmologist, with a specificity of 99.18 % (95% CI: 98.34–99.83%)

Among the six images with false positive VTDR classifications, in three images, the discrepancy was attributed to differentiating hard exudates from drusen. One image exhibited a pronounced RNFL reflex, which the model misinterpreted as macular edema. Another image contained hard exudates within one disc diameter of the fovea; however, the clinician’s grading placed the lesion outside the 1DD threshold. Representative examples are shown in Figure 3d.

The PPV was 96.21% (95% CI: 92.67–90.21) for vtDR. In contrast, the NPV is the proportion of eyes correctly identified as not having mtmDR or vtDR by the ophthalmologist’s grading among those classified as negative by AI, and it was 99.34% (95% CI: 92.67–99.21) for vtDR (refer to Table 1).

### 3.3. Performance of AI on DME

**Figure 4:**
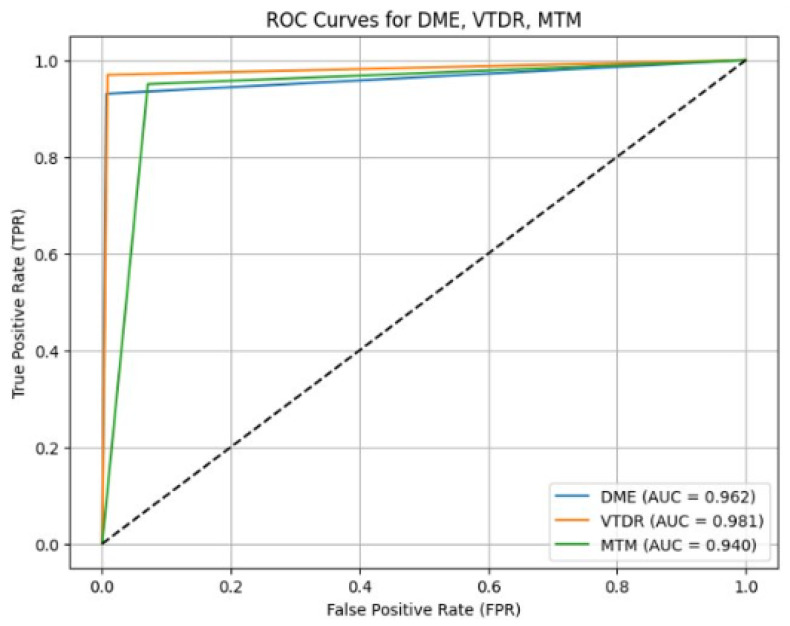
Receiver operating characteristic (ROC) curves for DME, VTDR, and MTM classification performance.

For the subset of images with DME, the AI system correctly identified 107 of 115 images that were identified as DME positive by the ophthalmologist, with a sensitivity of 93.04% (95% CI: 88.42–97.34%), and 620 of 624 images identified as DME negative by the ophthalmologist, with a specificity of 99.36 % (95% CI: 98.70–99.84%) with AUC 0.962 (refer to Figure 4).

**Figure 5:**
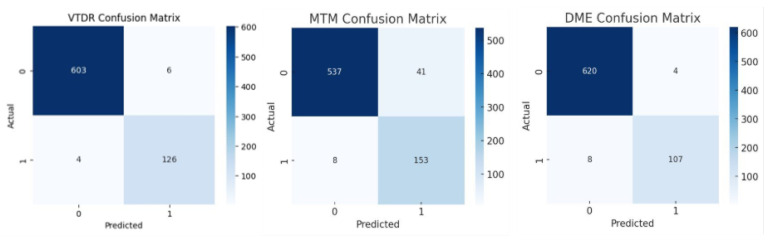
Confusion matrix of AI on mtmDR, VtDR, and DME.

In the analysis of the four false positive DME images (refer to Figure 5 for the confusion matrix), the discrepancy reflected the difficulty in distinguishing drusen from hard exudates in two cases. In the study of the eight false negative DME images, the discrepancy reflected the difficulty in identifying solitary and subtle hard exudates in five cases.

PPV, indicating the percentage of eyes with a positive AI result and the ophthalmologist grading as having DME present, was 96.04 (95%CI (92.38–99.2%).

NPV, which indicates the percentage of eyes with negative AI results and ophthalmologist grading as absent for DME, was 98.73 (95% CI (92. 38–99. 2%) (refer to Table 1).

### 3.4. Performance of AI Across Camera Types

The performance of the AI system across different fundus cameras is summarized in Table 2.

Sensitivity and specificity were comparable across cameras, with the AI system achieving the highest sensitivity on images from the Crystal Vue NFC camera, while it demonstrated slightly lower performance on images from the Canon CR2/CR2 AF. Despite minor variations, the AI system maintained consistent diagnostic accuracy across all camera types, indicating robustness to differences in image acquisition.

**Table 2:** Performance of the AI system across the camera types.

Camera Name	DR/DME	Sensitivity (%) (95% CI)	Specificity (%) (95% CI)	AUROC
Canon CR2/CR2 AF Total n = 192	mtmDR	93.62 (85.99–100)	86.86 (81.3–92.19)	0.90
	vtDR	96.55 (88.5–100)	98.06 (95.5–100)	0.97
	DME	95.83 (86.35–100)	98.75 (96.89–100)	0.97
Crystal Vue NFC 600/700 Total n = 190	mtmDR	100 (100–100)	100 (100–100)	0.99
	vtDR	100 (100–100)	99.32 (97.58–100)	1.00
	DME	96.88 (90.31–100)	100 (100–100)	0.98
Topcon NWC 400 Total n = 191	mtmDR	93.18 (85–100)	86.21 (79.99–91.54)	0.89
	vtDR	93.94 (84–100)	98.72 (96.66–100)	0.96
	DME	87.5 (74.28–97.30)	98.73 (96.81–100)	0.90
Zeiss VISUCAM 500 Total n = 187	mtmDR	94.12 (84.61–100)	98.67 (96.64–100)	0.94
	vtDR	96.97 (90–100)	100 (100–100)	0.98
	DME	92.59 (80.64–100)	100 (100–100)	0.96

## 4. Discussion

### Summary of Key Findings

In this retrospective clinical validation study, the AI system (version 2.1) demonstrated a high degree of precision, evidenced by its superior sensitivity and specificity in the identification of mtmDR, vtDR, and DME. Furthermore, the performance of the system remained uniform across various fundus cameras, highlighting its capacity for application in a wide variety of clinical settings.

In a meta-analysis of 10 studies using deep learning-based algorithms for the screening of DR in primary care, Wewetzer et al. reported a pooled sensitivity of 87% and a pooled specificity of 90% for the detection of any DR [9]. Upon evaluating deep neural networks for the screening of DR, Wang et al. reported a pooled sensitivity of 95% (95% CI: 93.0–97.0%) and a pooled specificity of 91% (95% CI: 88.0–93.0%) for more-than-mild DR (mtmDR), with a pooled sensitivity of 97% (95% CI: 94.0–99.0%) and a pooled specificity of 94% (95% CI: For vtDR [10]. The performance of our AI system is comparable to the benchmarks identified in these studies, many of which are now available as commercially deployed, regulatory-approved screening tools for DR.

Most studies have grouped DME within referable DR, but we analyzed it separately as an independent outcome. Although OCT remains the definitive tool for DME diagnosis, colour fundus photography is a more feasible modality for widespread screening. In colour fundus photos, DME can be identified by observing macular thickening through stereoscopic image pairs (ETDRS definition of CSME) or by using hard exudates within 1DD of the macula as a surrogate marker. Relying on fundus photography can lead to underdetection of DME, as noted in the pivotal IDx-DR trial, where the grading center missed 68% of cases involved with the OCT-proven DME [11].

A meta-analysis of 53 studies by Manikandan et al. evaluating deep learning models for the detection of DME using fundus images alone reported a pooled sensitivity of 94% (95% CI: 0.90–0.96). Accuracy varied substantially, ranging from 72% to more than 98%, while specificity was not consistently reported [12]. Concordant with this observation, our AI model demonstrated a sensitivity of 93.04% and a specificity of 99.36% when identifying DME, a performance that compares favorably with other similar AI algorithms that report sensitivities of 87–97% and specificities of 69–97% [13].

An AI system’s robust performance across commonly used fundus cameras is critical for meeting real-world requirements and inherent heterogeneity in practice environments. The performance of an AI system depends on the quality of the training data, with optimal results achieved when the dataset includes images from the same camera models used for screening. However, this presents a significant challenge for AI providers, as they optimize their software for a single, proprietary camera due to financial constraints, thus limiting its clinical utility. Two key studies highlight the real-world implications of this issue. Tufail et al. reported that while one AI system performed robustly across five different cameras, another showed statistically significant variability, with detection rates for PDR dropping from 100% on some camera images to 80% on another [14]. In their extensive study of over 102,856 images, Doğan et al. similarly evaluated a single AI system (across three different cameras) and confirmed that diagnostic sensitivity and specificity can vary significantly depending on the hardware used [15]. They further observed that, at the time of their study, most AI systems were not explicitly indicated for multi-camera use. By intentionally training on a diverse dataset from various camera models, our AI system achieves consistent and reliable performance that meets the requirements for real-world implementation, allowing clinics to integrate the system into their existing imaging infrastructure without being constrained by vendor-specific limitations.

Our decision to use an experienced clinician to grade single-field fundus images as the reference standard was intentional, helping to reflect real-world point-of-care screening conditions. This choice recognizes the distinction between the gold standard ETDRS classification used in reading center, academic, or clinical trial settings and the practical ICDR framework widely adopted in routine practice and screening programs. As noted by Scanlon et al., a key challenge for graders is distinguishing microaneurysms from hemorrhages on fundus photographs [16]. This challenge can result in under- or over-grading, as even a single hemorrhage, rather than microaneurysms alone, can change the final mtmDR grading from negative to positive, or vice versa, thus directly impacting AI performance in validation studies. In our analysis, this hemorrhage–microaneurysm discordance was identified as a primary source of discrepancies in the subset of images with mtmDR. Additionally, discordance arose from differentiating drusen from hard exudates, determining whether a hard exudate was within the 1-disc-diameter threshold, and the subjective assessment of the presence of venous beading. Notably, two of the four false negative images were still flagged as referable, indicating that the algorithm would have referred them, even in the presence of misclassification. In real-world settings, these features are often evaluated by clinicians rather than strictly structured grading criteria.

## 5. Strengths of the Study

This study has several key strengths that affirm the validity and real-world applicability of its findings. First, the study used a multi-camera dataset sourced from different sites, generating a diverse dataset of 739 analyzable images; the dataset was intentionally enriched with 40% of images that have DR features, allowing for a rigorous evaluation of sensitivity across the full spectrum of the disease. Second, we conducted dedicated analyses for DME and for each of the four individual camera models. Third, the reference standard was established by an experienced ophthalmologist using the International Clinical Diabetic Retinopathy (ICDR) Severity Scale, mirroring real-world screening workflows and enhancing the clinical relevance of the findings.

## 6. Limitations of the Study

The study also has limitations, such as a single-grader reference standard, a lack of demographic or clinical metadata, image-level rather than eye-level analysis, the absence of OCT confirmation of DME, and the inability to analyze the influence of mydriasis on image quality.

## 7. Conclusions

The DRISTi AI system (Version 2.1) demonstrated strong diagnostic performance for mtmDR, vtDR, and DME. This performance is not only comparable to leading commercially available AI systems for overall DR detection, but is unique in its consistent accuracy across multiple, commonly available fundus cameras. This capability ensures its seamless and reliable integration into diverse clinical imaging infrastructures, giving it a distinct advantage for comprehensive DR screening programs. A real-world prospective trial (NCT07222293) is ongoing and registered with the FDA.

## Figures and Tables

**Table 1:** Performance of AI on mtmDR, vtDR, and DME.

DR Grading/ Performance Criteria	mtmDR	vtDR	DME
Sensitivity (%) (95% CI)	95.03 (91.06–97.89)	96.95 (94.02–99.28)	93.04 (88.42–97.34)
Specificity (%) (95% CI)	92.91 (91.06–97.89)	99.18 (98.34–99.83)	99.36 (98.70–99.84)
PPV (%) (95% CI)	78.87 (72.86–84.57)	96.21 (92.67–99.21)	96.40 (92.38–99.20)
NPV (%) (95% CI)	98.53 (97.42–99.44)	99.34 (98.64–99.83)	98.73 (97.85–99.52)

## Data Availability

The data that support the findings of this study is not publicly available due to ethical reasons and is available upon request from Pradeep Walia (email: pwalia@artelus.com).

## Abbreviations

The following abbreviations are used in this manuscript:

AI	Artificial Intelligence
CNN	Convolutional Neural Network
CSME	Clinically Significant Macular Edema
DME	Diabetic Macular Edema
ETDRS	Early Treatment Diabetic Retinopathy Study
DR	Diabetic Retinopathy
mtmDR	More-Than-Mild Diabetic Retinopathy
NPDR	Non-Proliferative Diabetic Retinopathy
PDR	Proliferative Diabetic Retinopathy
vtDR	Vision-Threatening Diabetic Retinopathy
